# Systematically Prioritizing Functional Differentially Methylated Regions (fDMRs) by Integrating Multi-omics Data in Colorectal Cancer

**DOI:** 10.1038/srep12789

**Published:** 2015-08-04

**Authors:** Huihui Fan, Hongying Zhao, Lin Pang, Ling Liu, Guanxiong Zhang, Fulong Yu, Tingting Liu, Chaohan Xu, Yun Xiao, Xia Li

**Affiliations:** 1 College of Bioinformatics Science and Technology, Harbin Medical University, Harbin, Heilongjiang 150086, China

## Abstract

While genome-wide differential DNA methylation regions (DMRs) have been extensively identified, the comprehensive prioritization of their functional importance is still poorly explored. Here, we aggregated multiple data resources rooted in the genome, epigenome and transcriptome to systematically prioritize functional DMRs (fDMRs) in colorectal cancer (CRC). As demonstrated, the top-ranked fDMRs from all of the data resources showed a strong enrichment for known methylated genes. Additionally, we analyzed those top 5% DMR-coupled coding genes using functional enrichment, which resulted in significant disease-related biological functions in contrast to the tail 5% genes. To further confirm the functional importance of the top-ranked fDMRs, we applied chromatin modification alterations of CRC cell lines to characterize their functional regulation. Specifically, we extended the utility of the top-ranked DMR-coupled genes to serve as classification and survival biomarkers, which showed a robust performance across diverse independent data sets. Collectively, our results established an integrative framework to prioritize fDMRs, which could help characterize aberrant DNA methylation-induced potential mechanisms underlying tumorigenesis and uncover epigenome-based biomarkers for clinical diagnosis and prognosis.

Cytosine DNA methylation is a vital factor in genomic modification, which functions in the transcriptional silencing and epigenetic regulation of endogenous genes^1^. Since then, DNA methylation analysis has focused on identifying differentially methylated regions (DMRs) between different biological conditions. Specially, DMRs have been related to genomic imprinting, the control of tissue-specific genes during differentiation^2^,^3^, and a complex interplay with versatile regulatory elements to affect gene expression^4^ in diverse diseases^1^,^5^. Nevertheless, the genome-wide identification of DMRs with functional importance is still lacking.

To date, DMRs have been largely identified and extensively studied; however, whether all DMRs can give rise to functional alterations remains unknown. Differential DNA methylation may contribute to gene expression alterations, and there is great interest in the correlation between gene promoter methylation and inhibited expression^6^. However, in practice, the genome-wide correlation between DNA methylation and gene expression is approximately −0.3^7^,^8^. A highly nonlinear relationship between DNA methylation and gene expression is observed, with high DNA methylation levels generally associated with low expression, while low DNA methylation levels are associated with both high and low expression. For instance, DNA methylation-induced silencing of BRCA1 has been reported by The Cancer Genome Atlas (TCGA) breast cancer^9^ and ovarian cancer study^10^. HOXA methylation and differential expression in breast cancer has also been widely reported^11^. Meanwhile, Simmer *et al.*^12^ observed a preference of DMRs for genes either not or lowly expressed in normal colon tissue, which likely supports the existence of DMR sites with less functional importance. Additionally, the genome-wide distribution of DMRs is poorly understood, as a consistent preference for any genomic location is absent^13^,^14^. Evidence of altered gene expression could provide a glimpse into the functional importance of DMRs, therefore highlighting the desperate need for systematic prioritization of functional DMRs (fDMRs).

Based on the complex interplay between genomic and epigenetic modification of DNA methylation^15^, we reviewed many other involved factors in addition to the expression alterations of DMR-coupled genes, which could also be applied to prioritize fDMRs via serving as effective indicators of functional genomic regions^16^. Sequence conservation is now an accepted criterion to annotate genomic functional regions. As demonstrated by previous work, methylation is conserved among most eukaryotes, mainly in CG dinucleotides. Specifically, Eckhardt *et al.*^17^ indicated that regions with evolutionarily conservation are preferred sites for differential DNA methylation using bisulfite DNA sequencing. Clear evidence also shows that gene body methylation is conserved in most organisms, especially in exons^18^,^19^, which could therefore be an indicator of the functional importance of DMRs. Moreover, diverse regulatory elements^1^,^20^, such as transcription factor binding sites (TFBS), enhancers and insulators, are sensitive to DNA methylation alterations. These elements may establish and maintain functional regions in the genome, thereby affecting gene expression or repression^21^,^22^. For example, Bell *et al.*^23^ demonstrated that the occurrence of DNA methylation at an insulator controls the expression of the imprinted gene IGF2. As recently reported, the gene IGF2 contains DMRs that act both as methylation-sensitive silencers^24^ or activators^25^. Moreover, the dynamics of DMRs charted by Ziller *et al.*^26^ across different human tissues and cell lines also highlight the generalized functional importance of DMRs. Notably, fDMRs could affect cellular and molecular functions through multiple layers of regulation, and the functional importance of DMRs could be comprehensively characterized by integrating data from the genome, epigenome and transcriptome.

Here, we propose an integrative framework (Fig. 1), which combines gene expression alterations, conservation, genomic features of TFBS, DNaseI-hypersensitive sites (DHS), enhancers and insulators, and methylation dynamics across diverse tissues and cell lines to systematically prioritize fDMRs in colorectal cancer (CRC). In our results, literature-supported methylated genes in CRC were significantly ranked towards the top. Based on another set of DMRs computed from colon adenocarcinoma (COAD) methylation data obtained from the TCGA data portal, we re-ranked the COAD DMRs using our integrated prioritization approach, which showed a significant overlap between those top-ranked DMRs from our original rank and the new rank. Together with functional enrichment analysis, we demonstrated that top-ranked fDMR-coupled genes tend to play important roles in disease status. By combining data from the cancer cell lines Caco2 and HCT116, we observed that top-ranked fDMRs showed significant alterations of chromatin modifications, thus highlighting their functional importance. Finally, we showed that the top-ranked fDMRs could be applied as robust epigenome-based biomarkers for disease classification and serve as prognostic factors for CRC patients, across diverse independent data sets.

## Results

### Systematic prioritization of fDMRs

We obtained a published set of 2,680 DMRs in CRC from Simmer *et al.*^12^ and then analyzed the general features of those regions. As shown in Fig. 2A, the mean length of these DMRs is approximately 5091.80 bp. In comparison with randomly generated regions, those DMRs tend to overlap with CpG islands (CGI) and the first exons of coding transcripts with a significant enrichment ratio of 17.33 and 13.22, respectively, followed by the 5′ UTRs, CGI promoters and CGI shores of coding transcripts (Fig. 2B). Notably, the enrichment ratio of promoters of coding transcripts was unexpectedly low, which indicates that there might be a potential bias in associating a DMR with the promoter of a gene. Therefore, we considered DMRs falling within 100-kb flanking regions of genes (contains both coding and noncoding genes). Based on the relative locations between DMRs and genes, DMRs were classified as gene-overlapping, gene-proximal, gene-distal (10 kb), and gene-distal (100 kb) (details in Materials and Methods). Based on the proportion distribution of the four kinds of DMRs, we showed that most DMRs fell into coding genes, while the most overlapped with the 3′ flanking regions of noncoding genes (Fig. 2C). While, DMRs extensively covered both coding and noncoding genes, different proportions were observed per chromosome (Fig. 2D).

**Figure 1:**
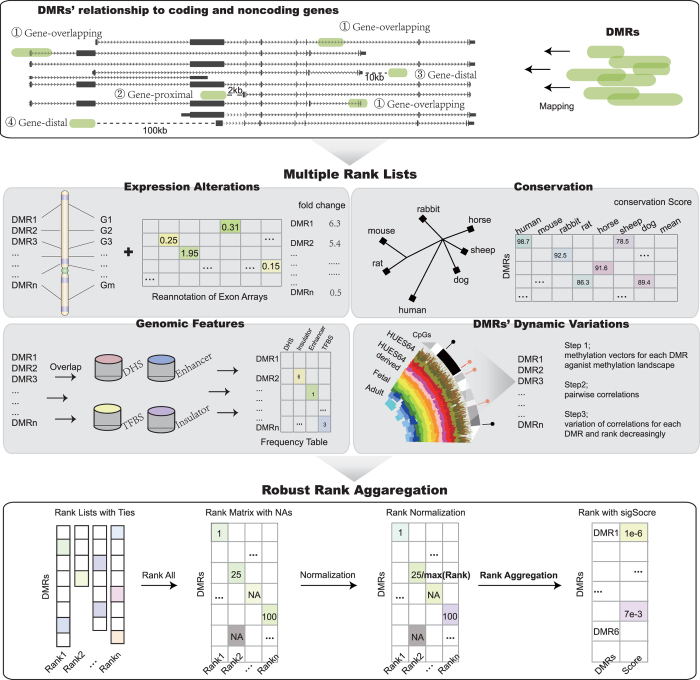
An integrative computational workflow for systematic prioritization of fDMRs. In total, three steps are involved in the prioritization process. Step 1. DMRs are filtered, and those coupled with at least one gene (coding or noncoding) are retained. Step 2. Based on expression alteration, conservation, genomic features and dynamic variations of DNA methylation, DMRs are ranked to generate individual ranks. Step 3. All individual ranks are aggregated based on order statistics.

**Figure 2:**
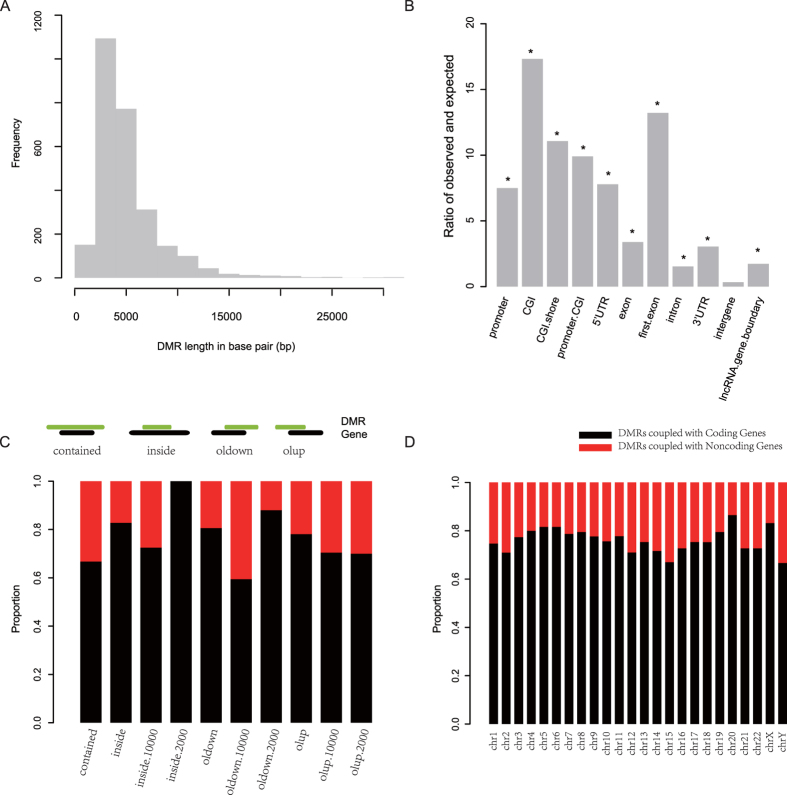
General characteristics of DMRs. Length frequency distribution (**A**) and fold enrichment ratios against different genomic elements (**B**) are shown. Moreover, by classifying those DMRs based on their coupled genes as coding or noncoding, the proportion distribution is plotted against different types (**C**) and chromosomes (**D**). The red bar indicates DMRs coupled with noncoding genes, while black bar represents DMRs coupled with coding genes. contained, DMR contains its coupled genes; inside, DMR is contained by genes; oldown, DMR overlaps with the downstream of the gene; olup, DMR overlaps with the upstream of the gene.

**Figure 3:**
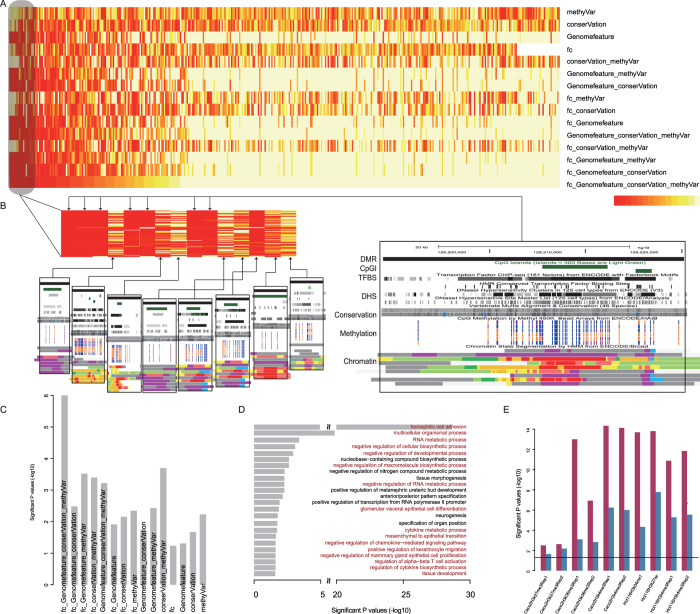
Validation of the aggregated rank based on multiple omics data. The heatmap shows relative ranks for all DMRs across all 15 possible ranks (**A**). DMRs are ordered according to the rank aggregated from all data resources. The color red indicates higher relative ranks. All DMRs ranked to the first position in each possible aggregated rank are also shown (**B**) with the UCSC genome browser plot in the black box. Prioritized features, such as TFBS, and conservation and dynamics of DNA methylation in DMRs are provided in browser pictures. Functional elements defined by chromatin segments are also exhibited. The ranks of DMRs were re-mapped to known methylated CRC genes, and then the significance of the average relative rank in contrast to the random distribution for all possible ranks was determined (**C**). The y-axis shows the minus log-transformed p values. Functional enrichment result with GO for the top 5% DMR-coupled coding genes is plotted with significant p values on the x-axis (**D**). The red bar represents significant GO terms tightly associated with disease status. Validations characterized by chromatin modification alterations are also plotted with minus log-transformed p values on the y-axis (**E**). The red bars indicate comparisons between top- and tail- ranked 5% DMRs, while the blue bars compare the number of significant DMRs between top- and tail-ranked 5% DMRs. The black line indicates the significance cutoff of 0.05.

To comprehensively prioritize fDMRs identified through genome-wide DNA methylation screens, we applied a computational framework to explore their functional importance using multiple data resources (Fig. 1) containing expression alterations of DMR-coupled coding and noncoding genes, conservation, occupancies of genomic features, and the dynamics of DNA methylation across diverse human tissues and cell lines (see Table 1). Then, 2,670 DMRs were prioritized to characterize their functional importance, requiring that each DMR be coupled with at least one coding or noncoding gene. Individual ranks generated from different features were combined based on order statistics^27^. As a result, we acquired 15 rank lists aggregated from all of the possible combinations of different individual ranks (Fig. 3A).

### Detecting the contributions of features to the comprehensive prioritization of fDMRs

There were a total of 666 DMRs in the top 5% of all of the rank lists, while only 11.3% of DMRs recurred in half of these rank lists. Accordingly, four DMRs were ranked among the top 5% in more than 10 rank lists. To evaluate the contribution rates of those features, rank correlations between the full-aggregated rank (based on all the individual ranks) and four individual ranks were computed using Pearson correlation. We showed that the individual rank from genomic features most highly related to the full-aggregated rank with a coefficient of 0.714, followed by features of conservation, fold changes of DMR-coupled genes and the dynamics of DNA methylation. One possible explanation for this result could be that the genome-wide distributions of DMRs give rise to difficulties in characterizing functional importance by only considering the nearest coding genes. We therefore included the occurrence of diverse regulatory elements (i.e., genomic features) across the human genome to prioritize those fDMRs, which showed a high concordance correlation with the full-aggregated rank. Because these genomic features contain TFBS, DHS, enhancers and insulators, we again correlated sub-individual ranks with the full-aggregated rank. As a result, we observed an approximately equal correlation of sub-individual ranks based on TFBS and DHS with the full-aggregated rank, showing a coefficient of 0.446 and 0.474, respectively. Meanwhile, the correlation of sub-individual ranks of insulators with the full-aggregated rank was approximately 0.383, while the smallest correlation coefficient of 0.207 occurred between the enhancer rank and the full-aggregated rank. As shown by the rank correlation between the rank from genomic features and sub-individual ranks, an elevated correlation of genomic feature integration was observed, demonstrating the need for feature integration in the rank prioritization^28^,^29^. Meanwhile, as shown by the correlations between individual ranks and the full-aggregated rank ranging moderately from 0.260 to 0.714, we reasoned that all of the features applied for uniform prioritization contributed to the final prioritized rank, which was integrated based on order statistics.

Moreover, we listed all of the top 1^st^ DMRs from all of the rank lists and showed corresponding features (Fig. 3B) with an additional exhibition of functional elements identified from chromatin segments across different cell lines. For example, the top 1^st^ DMR (chr3:128188852–128220273) recurred in 7 rank lists (Fig. 3B, right). Obviously, this region is filled with an enrichment of genomic features of TFBS and DHS. Additionally, features of conservation and the dynamics of methylation both contribute to the rank of the DMR. Evidence from functional regions generated from chromatin segments showed that this region contains areas covered by active and poised promoters (Fig. 3B, right, indicated by red and purple, separately). Moreover, our results showed high correlation coefficients between genomic features and the 7 rank lists (0.714, 0.804, 0.776, 0.783, 0.884, 0.887 and 0.871, respectively), which therefore indicates that the functional importance of the region might partly rely on its complex interplay with diverse regulatory elements and further induce alternative effects on gene expression or repression^30^,^31^.

### Functional validation of prioritized fDMRs

The prioritized DMR ranks aggregated from different individual ranks were validated using a known CRC methylated gene set (see Table 2) manually collected from approximately 2,000 published papers. We showed that the full-aggregated rank generated the smallest relative mean rank of 0.291 for the known gene set, with a significant p value less than 1e-6 (Fig. 3C, permutation test in Materials and Methods) in comparison with other part-aggregated (based on part individual ranks) or individual ranks. We concluded that the full-aggregated rank more accurately ranked the functional importance of those DMRs. Moreover, to validate the prioritized rank generated from our integrated approach, we again obtained COAD methylation data from the TCGA data portal to compute a new set of 3,067 DMRs. We annotated these regions against different genomic elements, which showed a significant enrichment in CGI shore, 3′ UTR and lncRNAs (Supplementary Fig. 1). After re-ranking those COAD DMRs, we applied a hypergeometric test to determine whether the original top-ranked CRC DMRs overlapped significantly with the top genes of the COAD DMR rank. As shown, we observed a significant recurrence between the top-ranked 5%, 10%, 20%, 30%, 40% and 50% of our original CRC DMR rank and top-ranked 5%, 10%, 20%, 30%, 40% and 50% of the new COAD DMR rank, with p values of 3.304e-06, 1.760e-09, 9.673e-15, 8.274e-10, 8.385e-11 and 2.9890e-10, respectively. Therefore, we chose the full-aggregated rank to prioritize fDMRs.

Moreover, focusing on the full-aggregated rank, we performed gene ontology (GO) enrichment analysis with a false discovery rate (FDR) less than 0.05, based on all coding genes coupled with the 5% top-ranked and tail-ranked DMRs. As a result (Fig. 3D), genes coupled with top-ranked DMRs tended to be enriched in functions potentially associated with the progression of CRC, such as homophilic cell adhesion^32^, mesenchymal to epithelial transition and negative regulation of chemokine–mediated signaling pathway. In contrast, those coding genes coupled with tail-ranked DMRs were not enriched for any GO categories. Furthermore, to confirm the functional importance of those top-ranked DMRs, we downloaded histone modification signals for the CRC cell lines Caco2 and HCT116. We reasoned that the genomic regions enriched for different chromatin modifications were functionally important. As demonstrated, the chromatin modifications H3K4me3, H3K27me3, and H3K36me3 in the cancer cell line Caco2, as well as H3K4me1, H3K4me3 and H3K27ac in HCT116 cells, were significantly different between these top- and tail- ranked DMRs using a Wilcox test (Fig. 3D). Moreover, based on randomly chosen regions, we identified DMRs significantly enriched for chromatin modifications among top- and tail-ranked DMRs. We then computed the difference in the number of significant DMRs between top- and tail-ranked DMRs using a chi-squared test. As shown for all chromatin modifications, the p values were less than 0.05 (Fig. 3E), suggesting the potential functional importance of these top-ranked DMRs.

**Figure 4:**
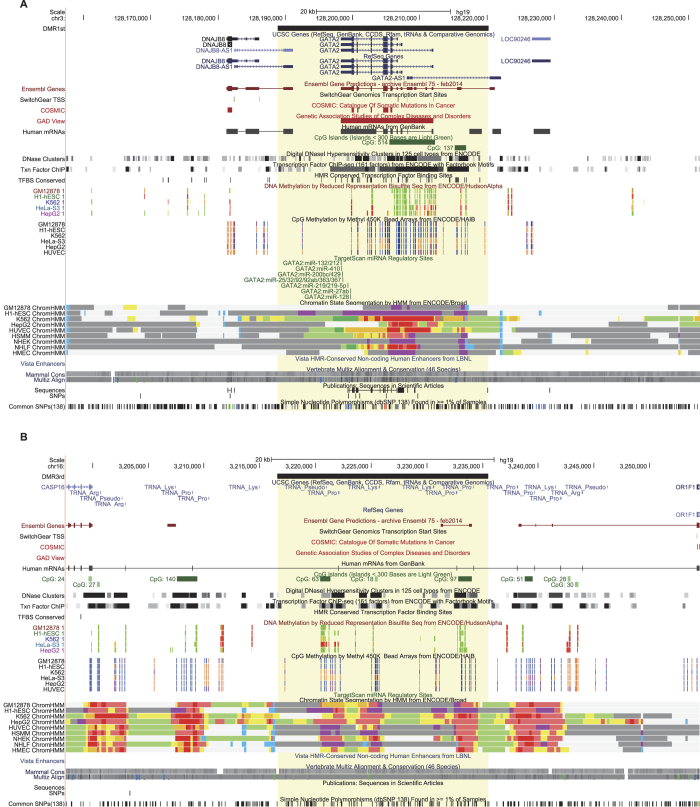
UCSC Genome Browser visualization for the top-ranked DMRs. Top 1st (**A**) and 3rd (**B**) DMRs are plotted with multiple tracks, such as their coupled coding and noncoding genes, mutations, DHS, TFBS, DNA methylation levels across diverse cell lines, miRNA-gene target relationships, and chromatin state segmentation by HMM and conservation, using the UCSC genome browser. The yellow shadow indicates the genomic region covered by the fDMR.

For example, the corresponding fDMRs of the known methylated genes vimentin (VIM) and secreted frizzled-related protein 1 (SFRP1) were ranked to the top 28^th^ and 142^nd^ positions, respectively. Nevertheless, we indicated that their aberrant methylation is critical underlying the progression of CRC. The gene VIM (Supplementary Fig. 2A) is hypermethylated at the promoter region and most of the gene body. Meanwhile, we observed co-enrichment for DNase I hypersensitivity sites and transcription factor binding sites from diverse cell lines compared with flanking regions. Based on the DNA methylation pattern across diverse cell lines measured by reduced representation bisulfite sequencing (RRBS) and Methyl 450 K Bead Arrays from The ENCODE Project Consortium, variability in the methylation levels of CpG sites included in the DMR of CRC was also observed, together with an increased conservation score for the entire region. As generally known, the protein encoded by VIM is involved in the immune response, whose abnormal activation is a high-risk factor underlying the progression of CRC^33^. Similarly, features characterizing the gene SFRP1 were also shown. As a member of the SFRP family, SFRP1 functions as a modulator of the Wnt signaling pathway. Moreover, its epigenetic silencing leads to dysregulation of the Wnt pathway^34^, which is tightly associated with CRC progression^35^,^36^. Additionally, we examined the top 1^st^ DMR, which completely covered gene GATA2. Although few studies have focused on GATA2 in the progression of CRC, we found that GATA2 is targeted by known CRC microRNA (e.g., hsa-mir-132^37^, has-mir-25^38^), resulting in decreased or silenced gene expression. As DNA methylation is tightly associated with gene silencing in diverse disease conditions^1^, this result implied a potential disease-related role for the GATA2 gene. We reasoned that the aberrant DNA methylation around GATA2 gene might represent an alternative dysfunctional regulatory event in addition to the post-transcriptional regulations of microRNAs. Moreover, we observed a significant difference in features such as DHS and conserved TFBS between regions covered by the fDMR and outside regions (Fig. 4A), which was similar to fDMRs related to those known methylated genes (see Supplementary Fig. 2). The top 3^rd^ DMR, is located within CpG dense regions, where a lot of tRNAs are also transcribed (Fig. 4B). Specifically, the fDMR is uniquely coupled with a long non-coding RNA (lncRNA) named AJ003147.8, which also co-occurs with a CGI, and is surrounded by other CGIs. Aberrant methylated CGIs have long been known to be involved in many tumor types^39^,^40^,^41^. Thus, we inferred that the lncRNA AJ003147.8 may be a potential candidate covered by an fDMR and might play an important role underlying the disease progression of CRC. However, due to the lack of related information in public databases, greater attention and additional efforts are required to study this lncRNA.

### Extended application of disease classification based on top-ranked DMRs

**Figure 5:**
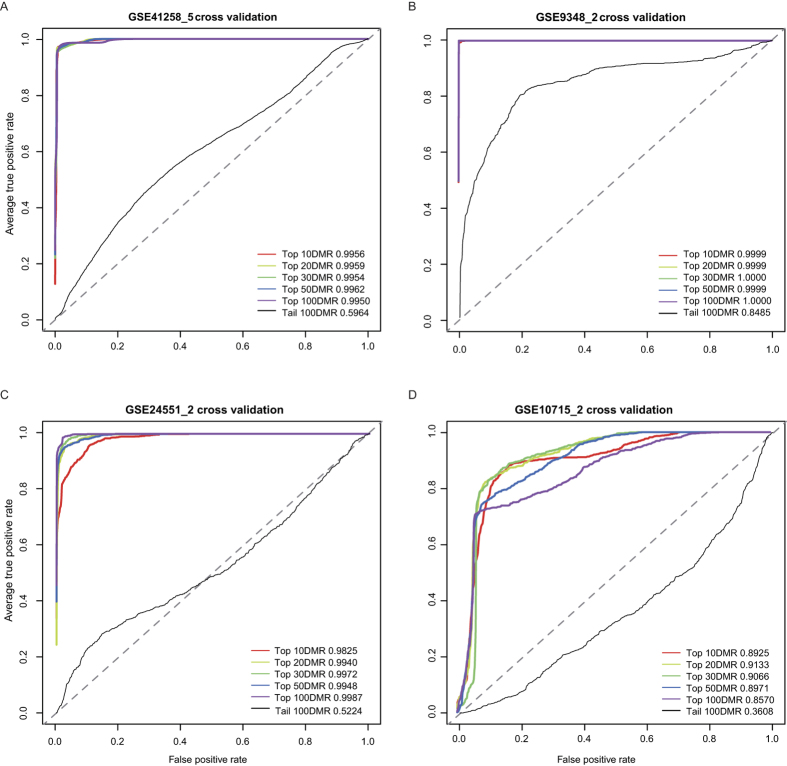
Classification analysis using epigenome-based gene sets. The relative operating characteristic (ROC) is used as a measure after 5- or 2-fold cross-validation for expression data sets GSE41258 (**A**), GSE9348 (**B**), GSE24551 (**C**), GSE10715 (**D**), separately. The ROC scores are shown in the corner of each plot, with the number of top- or down-ranked DMRs used as the signature set. As tail-ranked DMR-coupled genes are rarely represented on array chips, classification accuracies for tail-ranked 10, 20, 30 and 50 gene sets are missing.

Next, to explore whether these top-ranked fDMRs could serve as epigenome-based biomarkers in CRC, we applied their coupled coding and noncoding genes to distinguish patients from healthy controls (Fig. 5). Specifically, we applied the classification analysis to four previously published data sets (i.e., GSE9348, GSE41258, GSE10715 and GSE24551) involving 435 patients and 110 healthy controls (see Table 1). As demonstrated via microarray re-annotation, those top-ranked 10, 20, 30, 50 and 100 corresponding DMR-coupled gene sets (containing both coding and noncoding genes) could be used as predictive biomarkers to distinguish normal controls from patients. The relative operating characteristic (ROC) then served as a measure of the classification accuracy after cross-validation. As the number of normal samples varied across different data sets, we applied a two-fold cross-validation for the data set (i.e., GSE9348, GSE24551 and GSE10715) with normal samples around 10, and a 5-fold cross-validation for the remaining data set (i.e., GSE41258). For the expression dataset GSE41258, the accuracy of the five-fold cross-validation using the top epigenome-based gene sets ranged from 0.995 to 0.996 (Fig. 5A), while the tail-ranked DMR-coupled gene set failed to classify those samples at an accuracy of approximately 0.5, which is similar to random classifications (Fig. 5A; dotted line).

The accuracies of the two-fold cross-validation for dataset GSE9348, using the top epigenome-based gene sets, were approximately 1.000 (Fig. 5B), and the tail-ranked 100 DMR-coupled genes unexpectedly reached 0.849 (Fig. 5B). Moreover, we also used the exon expression dataset GSE24551 for classification validation. Similarly, the accuracies for top-ranked gene sets were approximately 0.99, while the tail-ranked genes only reached approximately 0.5 (Fig. 5C). Finally, a public expression dataset generated from peripheral blood was downloaded and then re-annotated. We observed excellent performance for these top-ranked epigenome-based gene sets, with accuracies ranging from 0.857 to 0.913, whereas a relative low accuracy (0.361) was observed for the tail-ranked gene set (Fig. 5D). Collectively, we suggest that these top-ranked DMRs tend to be functionally important in disease classification and could therefore be used as potential epigenome-based biomarkers.

### Clinical prognosis of top-ranked fDMRs in colon cancer

Based on the top-ranked DMR-coupled coding and noncoding genes, we further expanded our analysis to evaluate whether our full-aggregated DMR rank could identify survival-related epigenome-based marker genes. As CRC represents the third leading cause of death in cancers world-wide, CRC keeps challenging clinicians for significant diagnostic, prognostic and therapeutic tribulations. One recognizable feature of CRC is the difference in prognosis between early and late disease stages, with stage I-II patients showing a moderate risk of relapse after surgery and stage III patients showing a much higher risk of recurrence. Therefore, the identification of clinical risk factors tightly related with the survival time of stage III patients is critical.

Survival analysis was then conducted for each gene coupled with the top 5% DMRs using univariable Cox proportional hazards regression, according to the method of Kaplan and Meier. We retained those genes, that were correlated with disease-free survival time (DFS) of stage 3 patients in dataset GSE24551 (Supplementary Table 1) using a relatively loose cutoff of 0.1. As a result, a five-gene signature (Supplementary Table 2), including three coding genes (FOXC2, NKX2-2 and MATN4) and two long noncoding genes (HOXA-AS3 and GATA3-AS1), was identified. Then, we fitted these five marker genes into a multivariable Cox regression model to obtain their regression coefficients. Of these, one negative coefficient for MATN4 showed that its higher expression level was associated with longer survival. Conversely, the remaining four positive coefficients indicated that a higher level of expression (GATA3-AS1, NKX2-2, HOXA-AS3 and FOXC2) was related to decreased survival. Based on the expression of the five marker genes, we applied a risk score formula weighted by their regression coefficients for survival prediction, as follows: risk score = (−0.9704*expression level of MATN4) + (0.2665*expression level of GATA3-AS1) + (0.8831*expression level of NKX2-2) + (0.8831*expression level of HOXA-AS3) + (1.2354*expression level of FOXC2).

**Figure 6:**
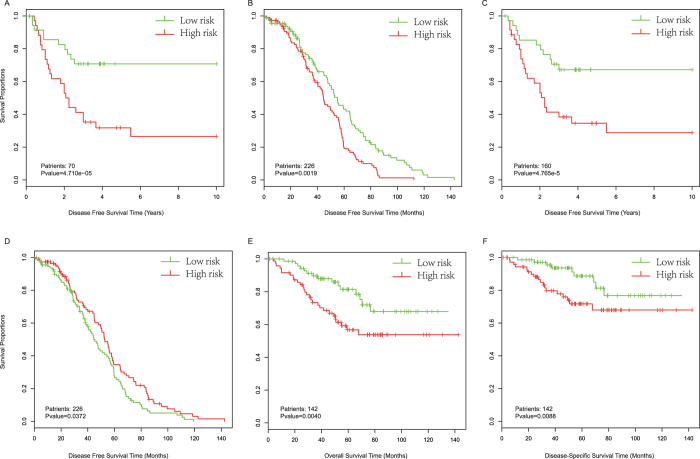
Kaplan-Meier estimates of the overall survival, disease free survival (DFS), or disease-specific survival (DSS) of GEO patients using epigenome-based marker genes. Patients were split into two groups based on their risk scores defines by marker genes. (**A**) Kaplan-Meier curves for GSE24551 stage 3 patients, based on marker genes coupled by top-ranked CRC DMRs. (**B**) Kaplan-Meier curves for GSE14333 patients as validation of the CRC marker genes. (**C**) Kaplan-Meier curves for GSE24551 stage 3 patients, based on marker genes coupled by top-ranked COAD DMRs. (**D**) Kaplan-Meier curves for the GSE14333 data set as a validation of marker genes from COAD. Kaplan-Meier curves for overall survival (**E**) and DSS (**F**) of GSE17536 patients, based on marker genes from COAD. Patient numbers and the significant p values are marked in the left corner. The differences between the two curves were determined by two-sided log-rank tests. The tick marks on the Kaplan-Meier curves indicate the censored subjects.

According to the risk formula, we were able to compute a risk score defined by the signature of each patient and then rank the patients. The patients were split into high-risk and low-risk groups, and our gene signature could significantly distinguish patients with long-term survival from those with short-term survival using the training data set (GSE24551, stage 3 patients), with a p value less than 1e-4 (Fig. 6A). Subsequently, we further verified the prognostic signature in another colorectal cancer data set (GSE14333). In accordance with the training data set, patients in the high-risk group showed a significantly shorter median DFS (log-rank test p = 0.0019, Fig. 6B). Because the data set GSE14333 also contained patient information regarding Duck’s stage, we again performed multivariable Cox regression analysis to include Duck’s stage as a covariant. As shown by the result, the risk score based on the five-gene signature remained significantly related to DFS even after adjustment according to Duck’s stage (log-rank test p = 0.0072).

To comprehensively evaluate the potential of our top-ranked DMR-coupled genes to serve as survival markers, we applied the entire survival analysis process to the DMR rank derived from the COAD data. Using the same parameters, we obtained four genes (Supplementary Table 2) that were significantly related to the DFS of patients in the training data set (log-rank test p < 1e-4). Additionally, we found that this signature demonstrated good performance for separating patients into high- and low-risk groups in the GSE24551 data set (i.e., combined both stage II and III patients, Fig. 6C). Consistently, the risk score-based classification yielded similar results for the DFS, overall survival and disease specific survival (DSS) of patients in other GEO data sets (Fig. 6D–F). In consideration of Duck’s stage in data set GSE14333, we adjusted the factor by running a multivariable Cox regression, which showed an insignificant log-rank p value when comparing the high- and low-risk groups. Similarly, we adjusted the AJCC stage in data set GSE17536 for overall survival and DSS. As shown, the risk scores of marker genes retained their significance associated with overall survival and DSS (log-rank p = 0.0001, and p = 0.0003, respectively). Collectively, with univariable and multivariable Cox regression analysis, we reasoned that our full-ranked DMR ranks based on multiple omics data indicated certain reference values, which may help to identify epigenome-based survival-related marker genes. However, further biological evaluations are still needed.

The coding gene FOXC2 and the long non-coding gene GATA3-AS1 are involved in the prognosis of CRC patients. FOXC2 belongs to the forkhead family of transcription factors, and this gene is significantly related to the degree of lymph node metastasis in CRC^42^ and might correlate with disease survival time. Additionally, this gene has been shown to serve as a novel prognostic factor in human esophageal squamous cell carcinoma^43^. LncRNA for GATA3-AS1 and GATA3 may be co-regulated by the same regulatory elements and thus show a similar expression level, with both pathways sharing functions in TH2 cell responses^44^ and T-cell development^45^. Indeed, the up-regulated expression of GATA3 in colitis^46^ is associated with an increased risk of CRC^47^. Moreover, the lncRNA GATA3-AS1 has been identified as a survival factor in COAD.

The coding gene NKX2-2 serves as a biological biomarker of multiple cancers, including Ewing sarcoma and diffuse Gliomas^48^,^49^, and this gene was also identified as a potential oncogene in T cell acute lymphoblastic leukemia^50^. The coding gene MATN4 encodes extracellular matrix protein found in various tissues and may be associated with wound healing^51^. Collectively, our results demonstrate that the coding genes NKX2-2 and MATN4 and the noncoding gene HOXA-AS3 may serve as novel survival factors in CRC.

## Discussion

Aberrant DNA methylation is strongly associated with cancer, and studies on DNA methylation aim to shed light on the progression of tumorigenesis underlying CRC, as well as to identify epigenome-based biomarkers for patient classification and survival analysis. In this study, we applied an integrated approach to systematically prioritize genome-wide DMRs based on their functional importance through the use of multiple resources including gene expression alterations, occupancies of genomic features, conservation, and dynamic variations of DNA methylation in DMRs across diverse human tissues and cell lines.

To confirm the necessity of integrating all individual ranks generated from different data resources, we listed all possible combinations of all individual ranks. By comparing the mean relative rank for the known methylated gene set collected manually, our results suggest that the rank aggregated from multiple data resources is more accurate than any other possible combinations. Moreover, we confirmed the functional importance of top-ranked fDMRs aggregated from all of the data resources using both GO annotations and chromatin modification alterations across cancer cell lines.

The correlation between DNA methylation levels and corresponding gene expression may be complex when the methylation is located within a gene body^52^,^53^ or intergenic regions^54^,^55^. Evidence also shows that a higher level of DNA methylation might be causally associated with increased gene expression levels^56^. Notably, functional methylation may cover part of the promoter^57^,^58^, 5' untranslated region (UTR)^17^, exons, introns^59^,^60^, 3'UTR^61^, as well as regions upstream^54^ and downstream of the gene. We therefore coupled a DMR with a gene when the DMR was located within the 100-kb flaking regions of a gene, rather than merely focusing on the promoter regions. Additionally, we reasoned that the extensive genome-wide distribution of DMRs might indicate a complex interplay with diverse genomic functional elements. In certain conditions, active transcription factors prefer to bind to methylated sites rather than unmethylated sites^62^,^63^, which suggests functional importance indicated by functional genomic elements, such as TFBS, DHS, enhancers and insulators. We therefore applied these functional elements to characterize the potential importance of each DMR. Moreover, we assumed that the dynamic variation of the methylation level within a DMR across diverse tissues and cell lines indicated that the DMR was actively regulated and potentially function-related. Regional conservation is also used as a measure of functional importance in sequence. Collectively, the functional importance of each DMR could be characterized by the accumulating effects of all features interacting with the methylated region, which therefore contributed to the systematic prioritization of fDMRs. However, as a result, we also concluded that the rank of certain DMRs could be dominated by only one or two features. With an increase in uncovered features involved in functions of DNA methylation, the prioritization of fDMRs might be enhanced by integrating additional features.

As an extension of clinical applications, we also applied our top-ranked DMRs to serve as epigenome-based biomarkers for disease classification. As described in our results, coding and noncoding genes coupled with top-ranked DMRs were useful in classification analysis. These DMRs could also be used as prognostic factors to distinguish between patients with long-term survival and those with short-term survival. We obtained a survival signature, containing two lncRNAs and three coding genes, which performed robustly across independent CRC data sets.

In conclusion, our developed framework for the systematic prioritization of fDMRs integrated multi-omics data resources. Moreover, based on classification and survival analysis, we showed that top-ranked fDMRs are biologically important and that their coupled coding and noncoding genes could serve as critical epigenome-based biomarkers.

## Materials and Methods

### Data resources

DMRs were collected from a published work by Simmer *et al.*^12^. These authors applied pair-wise comparisons of 24 matched cancer and normal colon tissues to compute DMRs based on a genome-wide DNA methylation analysis approach (MethylCap-seq)^64^, requiring high recurrence of these regions among all the comparisons. We then converted their genomic coordinates to the hg19 reference genomes and focused on 2,680 hyper-DMRs for further prioritization analysis.

Expression alteration: Exon-level expression profiling of 160 CRC patients and 13 normal samples was performed using an Affymetrix Human Exon 1.0 ST Array (GSE24551) with data downloaded from the National Center for Biotechnology Information (NCBI) data repository (see Table 1).

Conservation: Using the PhastCons program^65^, each nucleotide was assigned a conservation score. The program applies a hidden Markov model-based approach to estimate the probability of each nucleotide belonging to a conserved element, which has been previously derived from multiple alignments of 45 vertebrate genomes to the human genome (http://hgdownload.cse.ucsc.edu/goldenPath/hg19/phastCons46way).

Genomic features: Coding and non-coding gene coordinates of assembly hg19 were obtained from the GENECODE project (http://www.gencodegenes.org/). Other functional genomic elements included TFBS, DHS, enhancers and insulators. From the ENCODE Project Consortium, 236 ChIP-seq files for TFBS were downloaded (http://hgdownload.cse.ucsc.edu/goldenPath/hg19/encodeDCC/wgEncodeSydhTfbs/). DHS across 108 cell lines were extracted from the UCSC genome browser (http://hgdownload.cse.ucsc.edu/goldenPath/hg19/encodeDCC/wgEncodeUwDnase/); enhancers were extracted from the UCSC Vista track (hg19); and insulators were downloaded from the database CTCFBSDB (http://insulatordb.uthsc.edu/).

Dynamic variation: The DNA methylation landscape was charted by Ziller *et al.*^66^ based on 33 human tissues and cell lines using 45 whole-genome bisulfite sequencing (WGBS) data files. The DNA methylation level at single-CpG-site resolution was extracted between 0 and 1.

Independent methylation data set: The Illumina Infinium 450 k DNA Methylation data for COAD was downloaded from TCGA data portal (level 3; http://cancergenome.nih.gov), which includes 328 samples in total, with 290 COAD patients and 38 normal samples. The beta values (β values) were used to represent the methylation levels of each CpG locus detected per sample.

Known methylated CRC gene sets: Using the PubMed (http://www.ncbi.nlm.nih.gov/pubmed/) and PubMeth (http://www.pubmeth.org/) databases, we collected 13 known CRC-related genes with aberrant DNA methylation from approximately 2,000 papers following manual examination of whether those frequently methylated genes deposited in PubMeth were validated by biological experiments in PubMed. These CRC-related genes are frequently methylated and associated with CRC progression (see Table 2).

Chromatin modifications of cancer cell lines: Histone modification signals, including H3K4me3, H3K27me3, and H3K36me3 in the CRC cell line Caco2 and H3K4me1, H3K4me3 and H3K27ac in HCT116 cells, were downloaded from the ENCODE Project Consortium.

Data sets for classification analysis: To show whether these DMR-coupled genes could be applied for classification, we obtained another three public gene expression data sets (GSE9348, GSE41258 and GSE10715) from the Gene Expression Omnibus. These arrays were re-annotated using our in-house R scripts to generate the expression profiling of coding and noncoding genes. Together with data set GSE24551, all gene expression data sets were applied for patient classification.

Data sets for survival analysis: Two public expression data sets with survival time information (GSE14333 and GSE17536) were downloaded and re-annotated for survival analysis. Before that, data set GSE24551 with stage 3 patients was used to perform survival analysis for the identification of epigenome-based survival signature genes. Other related clinical information about these datasets is summarized in Supplementary Table 1.

All detailed data summaries are included in Table 1.

### Individual rank processing

DMRs were coupled to one coding or noncoding gene when the gene was located within the DMR or within its 2-kb, 10-kb, or 100-kb flanking regions. Subsequently, based on the position of the DMR to the transcribed units, DMRs were assigned to the following categories: gene-overlapping (overlapping with an annotated gene), gene-proximal (2 kb upstream of the annotated 5' end, and 2 kb downstream of the annotated 3' end), gene-distal (10 kb upstream and downstream of an annotated gene) and gene-distal (100 kb upstream and downstream of an annotated gene). DMRs with at least one coupled gene were used for further prioritization (Fig. 1).

The probe sets of Human Exon array (GSE24551) were re-annotated by mapping all probes to the human hg19 genome (hg19; http://www.gencodegenes.org/) using a customized R script. Those probes, which mapped uniquely to the genome with no mismatch, were kept. On the basis of the annotations from GENCODE, lncRNA genes with at least eight probes mapped uniquely to their exon sequences were obtained. The expression of lncRNA and coding genes was generated by RMA background correlation and quantile normalization. In total, the final re-annotated expression profiling contained 18,376 and 10,092 coding and noncoding genes, respectively. The absolute fold change of expression for each DMR-coupled gene in the CRC samples compared to normal samples was used as the measure of difference in expression. fDMRs are expected to have a high absolute fold change, thus with high ranks. Considering that several genes might be coupled with one DMR, we chose the largest absolute fold change of expression alterations to rank the region. As for the expression alteration feature, the rank was ordered with decreasing absolute fold change of the coupled genes. The average PhastCons conservation score was used to measure the conservation of DMRs. For the conservation feature, we ranked those DMRs on the basis of their decreasing conservation scores.

For genomic features including TFBS, DHS, enhancers and insulators, we first mapped each category of genomic features against those DMRs. The frequencies of enhancers overlapping with each DMR was computed and then ranked in a decreasing order. Like enhancers, the frequencies of insulators were also computed and ranked in the same way. For DHS, the narrow peaks from different cell lines were merged together to form a single peak set, which was then used to calculate the frequencies of DHS overlapping with each DMR. For TFBS, the frequencies of TFBS overlapping with each DMR in different cell lines were calculated and then summarized. Finally, the overlapping frequency table with four genomic features was ranked in decreasing order.

Based on the DNA methylation landscape characterized across diverse human tissues and cell lines, we calculated a variation score for each DMR, which represented the dynamic regulation of DNA methylation of DMRs under different conditions. The dynamic variation score was defined as the variance of pairwise Pearson correlations for each DMR. The correlations were then computed using the DNA methylation vector corresponding to the methylation level at single-base resolution to all the CpGs within the region across diverse conditions. Furthermore, all DMRs were ranked according to their dynamic variation score in decreasing order.

To fit the rank aggregation process based on sound order statistics, we applied relative ranks of each individual rank as the input to compute the final integrated rank.

### Rank aggregation based on order statistics

Regarding the individual ranks generated from the altered expression of the DMR-coupled genes, conservation, occupancies of different genomic features, and the dynamic variation score, we applied an integrated approach to aggregate all of the individual ranks into one final rank based on order statistics.

*n* represents the number of all individual ranks. Then, the rankings in each individual rank were divided by the maximal ranking, to obtain the relative rankings ranging from 0 to 1. A relative rank matrix was subsequently generated. ***r*** = (r_1_,…r_n_) represents the corresponding rank vector for a DMR, where r_j_ denotes the relative rank of the DMR in the j-th individual rank.

It was assumed that all of the relative ranks originated from a distribution strongly skewing toward zero, and our goal was to examine these distributions. Therefore, all of the ranks were sampled from a uniform distribution. For each individually normalized rank vector ***r***, r_(1)_,…,r_(n)_ represented an increasingly reordered rank vector, where *r*_(1)_≤,…,≤*r*_(n)_. We then calculated the probability to obtain 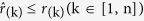 when 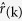 was generated from the null model.

β_*k,n*_ (***r***) represents the probability that 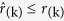. According to the null model, the probability that the order statistic 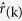 is smaller or equal to *x* can be computed from a binomial probability as follows:


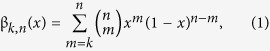


which means that at least *k* relative ranks must be in the range [0, *x*]. We therefore defined the final score for the rank vector ***r*** as the minimum of binomial probability


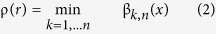


and order all of the rank vectors based on their ρ scores^27^. The method described above could successfully detect regions, which are consistently ranked more successfully than expected, and meanwhile assign a ranking score for each region. Moreover, the underpinning probabilistic model allows the algorithm to be not only parameter free but also robust to general outliers, especially noise and errors. We therefore applied the algorithm to integrate all of the individual ranks generated from different features.

### Identification of DMRs using microarray data

Based on the level 3 data from TCGA data portal, we applied the R package “ChAMP”^67^ to identify DMRs according to the probe lasso method^68^. ChAMP used dynamic windows incorporating probe association statistics (p-values) and genomic feature annotation (probe distribution) to identify significant aberrantly methylated regions. As required, significant probes needed to reach an adjusted p-value of 0.05 (Benjamini-Hochberg), and each DMR must contain at least three significant probes in the lasso. A lasso radius was defined as 2,000 bases, and the minimum DMR separation was defined as 1,000 bases according to the default parameters. Finally, a statistical threshold of FDR <0.05 was used to identify significant DMRs.

### Statistical analysis of known CRC methylated gene set based on random permutation

We re-mapped the ranks of DMRs to known CRC methylated genes, which were manually collected from the literature. Considering that there might be certain known methylated genes coupled with more than one DMR, the smallest rank of DMRs was used to represent the final rank of the gene. Subsequently, the average relative rank for the gene set was computed. To determine the statistical significance, a random permutation test was performed. We randomly selected the same number of genes as the real gene set 10,000 times to generate 10,000 random gene sets. We again re-mapped the ranks of all the random gene sets and then calculated the random average relative ranks. The significance level of the observed average rank was then defined as the probability of the times that those random average ranks were smaller than the observed one.

### Statistical analysis of histone modifications for cancer cell lines

The histone modification levels of each DMR were extracted using an in-house R script. We then sought to examine whether histone modifications among top- and tail-ranked DMRs showed significant differences. We directly compared the modification signals between top- and tail-ranked DMRs using the Wilcoxon rank sum test. As a complement, we randomly selected 1,000 random DMRs from those genomic areas measured by MethylCap-seq, keeping the same distribution of chromosome length and size as top- and tail-ranked DMRs, individually. Moreover, we required that the CpG density of all the randomly generated regions were comparable to those real regions^69^. Histone modification levels of random regions were then calculated. For each top- or tail-ranked DMR, the p value was calculated by counting the number of times that the histone modification levels of random regions were greater or lower than the observed value. Those DMRs with a p value of less than 0.05 were regarded as significantly different from random. Finally, we examined whether the number of significant top-ranked DMRs was significantly different from the tail-ranked ones using a chi-squared test.

### Survival analysis

Survival analysis was performed using the R package “survival” according to the method of Kaplan and Meier. The differences between the survival curves were assessed using the Log-rank test. To establish the association between top-ranked DMR-coupled genes (both coding and noncoding genes) and the DFS of patients, we applied univariable Cox regression analysis with a relatively loose cutoff of 0.1 to identify correlated genes. Those selected genes were then fitted into a multivariable Cox regression model to train their estimated regression coefficients. To construct a predictive model, we established a risk score formula, which included each of these selected genes weighted by their coefficients. According to this risk score, patients were reasonably dichotomized into high-risk and low-risk groups. Furthermore, survival differences between the two groups were assessed according to the log-rank test. We also applied our predictive model to another data set as a validation. To test whether the risk score was independent of Duke’s stage or AJCC stage, multivariate Cox regression analysis was again performed using the R package “survival”. Significance for all survival-associated comparisons was defined as a p value less than 0.05.

## Additional Information

**How to cite this article**: Fan, H. *et al.* Systematically Prioritizing Functional Differentially Methylated Regions (fDMRs) by Integrating Multi-omics Data in Colorectal Cancer. *Sci. Rep.* **5**, 12789; doi: 10.1038/srep12789 (2015).

## Supplementary Material

Supplementary Information

## Figures and Tables

**Table 1:** Multiple omics data used for rank aggregation and validation.

Data resources for rank aggregation:	
Expression relevance	Reannotation of exon microrarray (GSE24551)
Conservation Score	http://hgdownload.cse.ucsc.edu/goldenPath/hg19/phastCons46way/
Genomic Features	DNaseI hypersensitivity sites (DHS)
	http://hgdownload.cse.ucsc.edu/goldenPath/hg19/encodeDCC/wgEncodeUwDnase/
	Enhancer sites (UCSC Genome browser Vista Enhancer track)
	Insulator sites (insulator database; http://insulatordb.uthsc.edu/)
	Transcription factor binding sites (TFBS)
	http://hgdownload.cse.ucsc.edu/goldenPath/hg19/encodeDCC/wgEncodeSydhTfbs/
DMRs dynamic variation	45 whole genome bisulfite sequencing (WGBS) data sets across 33 diverse human cell and tissue types (GSE46644)
Data resources for rank validation:	
Classification analysis	ExpressionArray GSE9348 (case 70 samples; control 12 samples)
	ExpressionArray GSE41258 (case 186 samples; control 74 samples)
	ExpressionArray GSE10715 (case 19 samples; control 11 samples)
	ExonMicroArray GSE24551 (case 160 samples; control 13 samples)
Survival analysis	Disease-free survival analysis GSE24551 (stage 3; 70 patients)
	Disease-free survival analysis GSE14333 (226 patients)
	Overall/disease-specific survival analysis GSE17536 (142 patients)

**Table 2:** Known methylated genes manually collected from literature.

Gene name	Correspoding DMR
VIM	chr10:17270075–17272664
PCDH10	chr4:134067015–134075515
SFRP1	chr8:41165084–41169957
ADAMTS1	chr21:28215244–28220297
SLIT2	chr4:20252695–20257539
CDH4	chr20:59816829–59839645
SFRP2	chr4:154707902–154714964
HS3ST2	chr16:22823339–22827448
CHFR	chr12:133474262–133488352
SST	chr3:187386391–187389333
CDH13	chr16:82659883–82662617
MYOD1	chr11:17739468–17745225
TMEFF2	chr2:193057523–193061993
