# Supplementary material for: Systematically Prioritizing Functional Differentially Methylated Regions (fDMRs) by Integrating Multi-omics Data in Colorectal Cancer
Source: Sci Rep. 2015 Aug 4;5:12789. doi: 10.1038/srep12789 (PMC4523937; doi:10.1038/srep12789)
Supplement: Supplementary Information [file srep12789-s1.doc]

**Supplementary Information**

**Systematically Prioritizing Functional Differentially Methylated Regions (fDMRs) by Integrating Multi-omics Data in Colorectal Cancer**

Huihui Fan^&,1^, Hongying Zhao^&,1^, Lin Pang^1^, Ling Liu^1^, Guanxiong Zhang^1^, Fulong Yu^1^, Tingting Liu^1^, Chaohan Xu^1^, Yun Xiao^*,1^, Xia Li^*,1^

**Inventory of Supplementary Information**

1. Supplementary Figure 1

2. Supplementary Figure 2

3. Supplementary Table 1

4. Supplementary Table 2s

Supplementary Figure 1. Fold enrichment against different genomic elements.

**Supplementary Figure 1**. Fold enrichment against different genomic elements. Observed number was determined as the number of the real DMR set falling into per element. Random region sets from genomic areas measure by The Illumina Infinium 450k DNA Methylation microarray were obtained 1000 times, with similar length, chromosome and CpG density distribution as the real DMR set. A ratio between the observed number and mean of the 1000 random numbers was subsequently computed per element.

Supplementary Figure 2.


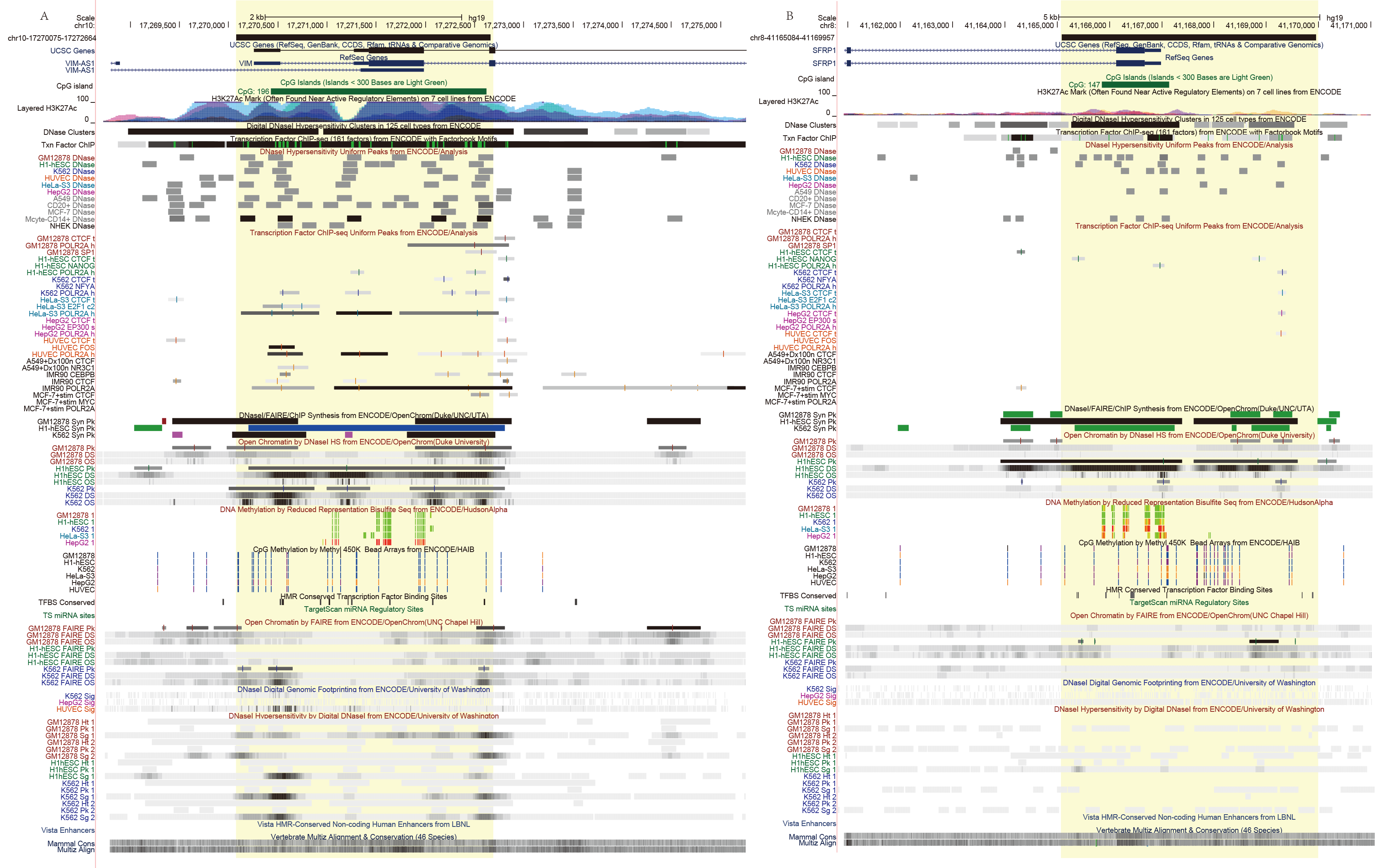


**Supplementary Figure 2.** UCSC Genome Browser visualization for known methylated CRC genes. Examples show multiple genomic features for gene VIM (A) and SFRP1 (B). Their coupled hyper-methylated regions for prioritization are indicated by black bar in the first track. Other related features, such as SNPs, COSMIC mutations, GAD genes, H3K27Ac mark distribution, DHS, TFBS, conservation, and DNA methylation levels across other tissues and cell lines are also included as custome tracks, using UCSC genome browser. Yellow shadow indicates the region covered by the hyper-methylated region identified in CRC.

Supplementary Table 1. Detailed information about data sets used for survival analysis.

| Clinical Information | | Data Sets used for Survival Analysis | | |
| --- | --- | --- | --- | --- |
|  |  | GSE24551 | GSE14333 | GSE17536 |
| Tumor Stage | Ⅱ | 90^a^ |  |  |
|  | Ⅲ | 70 |  |  |
| MSI Status | MSI-H | 21 |  |  |
|  | MSI-L | 16 |  |  |
|  | MSS | 110 |  |  |
|  | NA | 26 |  |  |
| Ducke’s Stage | A |  | 41 |  |
|  | B |  | 94 |  |
|  | C |  | 91 |  |
| Location | Colon |  | 2 |  |
|  | Left |  | 93 |  |
|  | Rectum |  | 30 |  |
|  | Right |  | 101 |  |
| AJCC Stage | 1 |  |  | 24 |
|  | 2 |  |  | 55 |
|  | 3 |  |  | 56 |
|  | 4 |  |  | 10 |

^a^ represents the number of samples in each clinical characteristics.

Supplementary Table 2. Epigenome-based marker genes.

|  | **Gene Symbol** | **Harzard Ratio** | **Coefficient** | **DMRs** |
| --- | --- | --- | --- | --- |
| **CRC-seq-bsed** | MATN4 | 0.379 | -0.9704 | chr20:43936958-43958173 |
|  | GATA3-AS1 | 1.3054 | 0.2665 | chr10:8089115-8106797 |
|  | NKX2-2 | 2.4185 | 0.8831 | chr20:21484513-21504818 |
|  | HOXA-AS3 | 1.2354 | 0.2114 | chr7:27191891-27195455 |
|  | FOXC2 | 4.8715 | 1.2354 | chr16:86597925-86617721 |
| **COAD-chip-based** | HOXC4 | 2.0202 | 0.7032 | chr12:54426559-54426886 |
|  | TLE3 | 0.8456 | -0.1677 | chr15:70390354-70390377 |
|  | GATA3-AS1 | 2.069 | 0.7271 | chr10:8094404-8096677 |
|  | ATF7 | 7.1234 | 1.9634 | chr12:54088778-54093258 |
